# Clinical-biological characteristics and treatment outcomes of pediatric pro-B ALL patients enrolled in BCH-2003 and CCLG-2008 protocol: a study of 121 Chinese children

**DOI:** 10.1186/s12935-019-1013-9

**Published:** 2019-11-14

**Authors:** Chao Gao, Shu-Guang Liu, Zhi-Xia Yue, Yi Liu, Jing Liang, Jun Li, Yuan-Yuan Zhang, Jiao-Le Yu, Ying Wu, Wei Lin, Hu-Yong Zheng, Rui-Dong Zhang

**Affiliations:** Beijing Key Laboratory of Pediatric Hematology Oncology, National Key Discipline of Pediatrics (Capital Medical University), Key Laboratory of Major Diseases in Children, Ministry of Education, Hematology Oncology Center, National Center for Children’s Health, Beijing Children’s Hospital, Capital Medical University, 56 Nanlishi Road, Beijing, 100045 China

**Keywords:** Pro-B cell, Pediatric, Acute lymphoblastic leukemia, Biological characteristics, Prognostic factor

## Abstract

**Background:**

Although leukemic blast cells of Pro-B cell acute lymphoblastic leukemia (ALL) are arrested at the same stage of B cell differentiation, the immature B cell subtype is still biologically heterogeneous and is associated with diverse outcomes. This study aimed to explore the clinical-biological characteristics of pediatric pro-B ALL and factors associated with outcomes.

**Methods:**

This study enrolled 121 pediatric patients aged 6 months to 14 years with newly diagnosed CD19^+^CD10^−^ pro-B cell acute lymphoblastic leukemia (pro-B ALL) treated at Beijing Children’s Hospital from March 2003 to October 2018. Genetic abnormalities, immunophenotypic markers, minimal residual disease (MRD) at early treatment stage and long-term outcomes of children treated on two consecutive protocols were analyzed.

**Results:**

*KMT2A* rearrangements were the most frequent abnormalities (incidence rate 33.06%), and were associated with lower frequency of CD13, CD33, CD22 and CD34 expression and higher frequency of CD7 and NG2 expression. Higher frequency of CD15 and CD133 expression was found in *KMT2A*-*AFF1*^+^ patients, exclusively. Presence of CD15 and absence of CD34 at diagnosis correlated with the high burden of MRD at the early stage of treatment. Outcomes were more favorable in patients older than 1 year, with absence of CD20 expression and *KMT2A* rearrangements, and with MRD lower than 1% at the end of induction and 0.1% before consolidation. Increased intensity of chemotherapy based on MRD analysis did not improve outcomes significantly (5-year EFS 73.9 ± 6.5% for BCH-2003 and 76.1 ± 5.3% for CCLG-2008, *P *= 0.975). Independent adverse prognostic factors were MRD ≥ 0.1% before consolidation and presence of *KMT2A* gene rearrangements (odds ratios [ORs] 9.424 [95% confidence interval (CI) 3.210, 27.662; *P *< 0.001]; 4.142 [1.535, 11.715, *P *= 0.005]; respectively).

**Conclusions:**

Pediatric pro-B ALL is a heterogeneous disease. Genetic analysis and MRD evaluation can predict patients with dismal prognosis; however, intensive chemotherapy alone does not improve outcomes of these patients and targeted therapy or hematopoietic stem cell transplantation may be required.

## Background

B cell precursor acute lymphoblastic leukemia (BCP-ALL) is the most frequent type of pediatric leukemia, representing about 80% of childhood ALL. Based on the stages of differentiation of normal B cell progenitors, BCP-ALL is classified into pro-B ALL (CD19^+^ CD10^−^ cyμ^−^ Igκ^−^ Igλ^−^), common-B ALL (CD19^+^ CD10^+^ cyμ^−^ Igκ^−^ Igλ^−^) and pre-B ALL (CD19^+^ CD10^+/−^ cyμ^+^ Igκ^−^ Igλ^−^) [1]. Pro-B ALL, accounting for 6% of pediatric and 16% of adult ALL [2, 3]. This is the most immature subtype of B-ALL and it originates from immature B cell precursors with maturational arrest at the pro-B cell stage. Pro-B ALL has been reported to be associated with unfavorable clinical features and poor prognosis in both childhood and adult patients [2, 4].

Although the leukemic blast cells of pro-B ALL are arrested at the same stage of B cell differentiation, the immature B cell lineage subtype of ALL is biologically heterogeneous and is associated with diverse outcomes [3]. *KMT2A* rearrangements are the most frequent genetic alteration in pro-B ALL, occurring in one-third of patients, with male prevalence and age less than 1 year; it is associated with dismal prognosis and aggressive clinical features, including hyperleukocytosis and central nervous system (CNS) involvement at diagnosis [2, 5]. High expression of Neuron-Glial antigen 2 (NG2), stem-cell antigen CD133, CD135, myeloid-associated antigen CD15 and CD65s, no expression of CD13, CD 33, and low expression of CD34 are found in *KMT2A*-*AFF1* positive patients [6–8]. Other genetic abnormalities, such as *BCR*-*ABL1*, have also been reported in about 9% of adult pro-B-ALL patients [3].

In current clinical practice, minimal residual disease (MRD) is assessed to determine the treatment response and subsequent prognosis in children with B-ALL. In many cohorts, MRD testing is introduced in the classification strategy and patients with high MRD levels receive more intensive chemotherapy [9]. In a multicenter clinical study of childhood ALL in China, better 5-year event-free survival (EFS) was achieved in an MRD adjustment group [10]. However, little is known about the significance of MRD in the pro-B ALL subset.

In the present study, we investigated the clinical features, genetic alterations, immunological characteristics, MRD at the early treatment stage and long-term outcomes in a large cohort of pediatric pro-B ALL patients in China treated with two consecutive protocols. Our objective was to further elucidate the biological heterogeneity of this immunophenotypic subgroup and the prognostic factors associated with patient outcomes.

## Methods

### Patients and treatment protocols

This study included 121 pediatric patients, 6 months to 14 years of age (median, 5 years) with newly diagnosed CD19^+^CD10^−^ pro-B cell acute lymphoblastic leukemia (pro-B ALL) treated at the Hematology and Oncology Center of Beijing Children’s Hospital, Capital Medical University, between March 2003 and October 2018. The BCH-2003 (Beijing Children’s Hospital-2003) and CCLG-2008 (Chinese Childhood Leukemia Group-2008) protocols were followed in 46 and 75 patients, respectively. The BCH-2003 protocol stratified patients into standard-risk (SR), intermediate-risk (IR) and high-risk (HR) according to their clinical and biological characteristics, prednisone response and morphological remission at the end of induction. Based on this stratification, the CCLG-2008 protocol administered MRD evaluation to modify treatment intensity, in which SR patients with day 33 MRD ≥ 0.01% and < 1% were upstaged to IR, and patients with day 33 MRD > 1% or week 12 MRD > 0.1% were upstaged to HR. Details regarding the stratification and treatment regimens for BCH-2003 and CCLG-2008 protocols are outlined in Additional file 1: Figure S1 and Additional file 2: Table S1, as previously described [11–13]. Both protocols were approved by the Beijing Children’s Hospital Institutional Committee. Signed informed consent was provided by the parents or guardians of each pediatric patient.

### Immunophenotyping

Patient mononuclear cells were isolated from pretreatment bone marrow aspirate samples by centrifugation on Ficoll-Hypaque density gradients, and stained with combinations of fluorochrome-conjugated monoclonal antibody panel (see Additional file 2: Table S2). Value of 20% was chosen as threshold of positivity for surface marker and 10% for intracytoplasmic antigen. Patients were classified as pro-B, common-B, pre-B, mature-B, T-cell and AML according to the criteria of the European Group for the Immunological Characterization of Acute Leukemias (EGIL) classification system [1]. Expression of CD19 and absence of CD10 surface antigen were required for the diagnosis of pro-B ALL and the immunophenotyping of one pro-B patient is shown in Additional file 3: Figure S2.

### Molecular and Fluorescence in situ hybridization (FISH) analysis

Fusion transcript analysis was performed by a multiplex reverse transcription polymerase chain reaction (RT-PCR), as described previously [11, 14]. This system was able to simultaneously detect 29 fusion transcripts associated with B-ALL including *BCR/ABL1*, *E2A*-*PBX1*, *E2A*-*HLF*, *ETV6/RUNX1*, *KMT2A/AFF1*, *KMT2A*-*MLLT1*, *KMT2A*-*MLLT3* and *KMT2A*-*MLLT10* which are related to B-ALL. Patients were also investigated by interphase FISH for *KMT2A* rearrangements using LSI KMT2A Dual-color Break-Apart Rearrangement Probe (Abbott Laboratories, Dallas, TX, USA). The detailed FISH procedure has been documented in a previous study [15]. FISH images of one *KMT2A* rearrangement-positive patient are presented in Additional file 4: Figure S3.

### Analysis of minimal residual disease

MRD was tested by flow cytometry and/or PCR quantitative method for immunoglobulin and T-cell receptor antigen gene rearrangements. Details of the performance and analysis of MRD by PCR were documented previously [11]. Leukemia-associated immunophenotypes were determined by multiparameter flow cytometry, using 11 panels of monoclonal antibodies conjugated to fluorescein isothiocyanate (FITC), phycoerythirin (PE), allophycocyanin (APC) and perdinin chlorophyII protein (PerCP). The immunophenotypic markers of MRD monitoring were shown in Additional file 2: Table S3 and one patient screening at diagnosis is shown in Additional file 5: Figure S4. For each patient, the marker combination of MRD was determined at diagnosis, identifying at least one leukemic cell per 10^4^ normal nucleated bone marrow cells. The procedures for cell collection, separation, staining and protocol for flow cytometric detection of MRD followed the guidelines of St. Jude Children’s Research Hospital, as described previously [16]. At least 1 × 10^5^ mononuclear cells were added into each MRD test tube. Bone marrow aspirates were collected at day 33 at the end of induction remission and day 78 before consolidation therapy. The leukemic involvement of < 0.01% of nucleated bone marrow cells was regarded as MRD negative [17]. Isotypical immunoglobulins were used as negative controls.

### Statistical analysis

The reference date for the end of data collection for the purpose of statistical analysis was December 31, 2018. Comparisons between patients with or without distinct immunological marker, *KMT2A* gene rearrangements and associations of pretreatment characteristics and response to treatment were evaluated by nonparametric tests. EFS was defined from the date of diagnosis to the date of relapse, death, or induction failure, whichever came first, or the last contact with patients in continuous hematologic complete remission. EFS distribution of patients with different clinical and biological features was estimated using the Kaplan–Meier procedure; comparisons between groups were performed using the log-rank test. The Cox proportional hazards regression model was used to determine significant differences in associations between patients’ biological/clinical indicators and outcomes. All tests were two-sided and *P* < 0.05 was considered statistically significant. SPSS 16.0 software (SPSS, Chicago IL, USA) was used for all statistical analyses.

## Results

### Genetic abnormalities in pro-B-ALL

Specific fusion transcripts were detected in 40 patients, with an incidence rate of 33.06% (40/121), involving *KMT2A* rearrangements, *BCR*-*ABL1*, *ETV6*-*RUNX1* and *E2A*-*PBX1*. The description of the incidence of each fusion transcript is shown in Additional file 2: Table S4. *KMT2A* gene rearrangements, accounting for one-quarter of pediatric pro-B-ALL patients, were the most frequent translocation. Compared with patients with *BCR*-*ABL1*, *ETV6*-*RUNX1* and without fusion, *KMT2A* gene-rearranged patients had a significantly higher proportion of infants and higher WBC counts. However, no differences were found in prednisone response and early treatment MRD between fusion subgroups (see Table 1). Furthermore, no differences were found in clinical and biological characteristics between patients with *KMT2A*-*AFF1* and other *KMT2A* rearrangements (see Additional file 2: Table S5).

**Table 1:** Correlation between specific fusion transcripts and clinical features of pro-B-ALL

Clinical features	*KMT2A* rearrangement (%)	*BCR*-*ABL1* (%)	*ETV6*-*RUNX1* (%)	Pro-B without fusion (%)	*P*
Gender					
Male	18 (56)	4 (100)	0 (0)	47 (58)	0.074
Female	14 (44)	0 (0)	3 (100)	34 (42)	
Age (years)					
< 1	9 (28)	0 (0)	0 (0)	2 (2)	*<* *0.001*
1–10	20 (63)	2 (50)	2 (67)	63 (78)	
≥ 10	3 (9)	2 (50)	1 (33)	16 (20)	
WBC					
< 50 × 10^9^/L	16 (50)	4 (100)	3 (100)	60 (74)	*0.027*
≥ 50 × 10^9^/L	16 (50)	0 (0)	0 (0)	21 (26)	
Prednisone response					
Good	29 (91)	4 (100)	3 (100)	75 (93)	0.835
Poor	3 (9)	0 (0)	0 (0)	6 (7)	
MRD at day 33					
< 0.01%	10 (37)	1 (25)	0 (0)	15 (24)	0.747
0.01–1%	14 (52)	2 (50)	2 (100)	35 (56)	
≥ 1%	3 (11)	1 (25)	0 (0)	12 (19)	
MRD at day 78					
< 0.1%	22 (81)	3 (100)	2 (100)	51 (86)	0.789
≥ 0.1%	5 (19)	0 (0)	0 (0)	8 (14)	

Italic signifies *P* < 0.05

### Immunophenotypic characteristics of pro-B ALL

All patients with pro-B-ALL were positive for CD19, cyCD79a and HLA-DR. 63.64%, and 6.31% of patients expressed B cell markers CD22 and CD20, respectively. T cell markers, including CD2, CD5 and CD7, were detected in fewer than 10% of patients, and no cytoplasmic CD3 antigen was detected. Myeloid lineage markers CD13 and CD33 were expressed in 20.66% and 50.41% of patients, respectively. Approximately four-fifths of patients demonstrated CD34 and cyTdT expression. Over one-third of patients expressed primitive cell antigen CD133, and only 2.48% patients expressed hematopoietic progenitor CD117. Aberrantly expressed CD15, CD56 and CD58 were detected in 21.05%, 15.79% and 12.40% of patients, respectively. Carcinoembryonic antigen CD66c was found in 6.58% and NG2 was detected in 39.47% of patients (see Additional file 2: Table S6). Further analysis of correlations between the expression of specific immunological markers and clinical features revealed that the frequency of NG2 expression was significantly higher in patients younger than 1 year old. Patients with CD15 expression had a higher ratio of MRD ≥ 1% at day 33 and ≥ 0.1% at day 78, while patients with CD34 expression had a lower proportion of MRD ≥ 1% at day 33 than those without (see Table 2). Meanwhile, no correlations were found between other immunological markers and clinical features (Additional file 2: Table S7).

**Table 2:** Correlation between immunological markers and clinical features of pro-B-ALL

Clinical features	CD15			CD34			NG2		
	Neg (%)	Pos (%)	*P*	Neg (%)	Pos (%)	*P*	Neg (%)	Pos (%)	*P*
Gender									
Male	35 (58)	9 (56)	0.881	14 (54)	55 (58)	0.712	27 (59)	17 (57)	0.861
Female	25 (42)	7 (44)		12 (46)	40 (42)		19 (41)	13 (43)	
Age (years)									
< 1	6 (10)	4 (25)	0.227	2 (8)	9 (9)	0.812	2 (4)	8 (27)	*0.012*
1–10	45 (75)	11 (69)		20 (77)	67 (71)		36 (78)	20 (67)	
≥ 10	9 (15)	1 (6)		4 (15)	19 (20)		8 (17)	2 (7)	
WBC									
< 50 × 10^9^/L	42 (70)	12 (75)	0.695	18 (69)	66 (69)	0.981	35 (76)	19 (63)	0.231
≥ 50 × 10^9^/L	18 (30)	4 (25)		8 (31)	29 (31)		11 (24)	11 (37)	
Prednisone response									
Good	54 (90)	16 (100)	0.188	24 (92)	88 (93)	0.956	41 (89)	29 (97)	0.234
Poor	6 (10)	0 (0)		2 (8)	7 (7)		5 (11)	1 (3)	
MRD at day 33									
< 0.01%	9 (17)	3 (19)	*0.042*	11 (26)	16 (21)	*0.001*	7 (18)	5 (17)	0.881
0.01–1%	38 (72)	7 (44)		10 (24)	43 (57)		26 (67)	19 (63)	
≥ 1%	6 (11)	6 (38)		21 (50)	16 (21)		6 (15)	6 (20)	
MRD at day 78									
< 0.1%	46 (94)	10 (67)	*0.005*	18 (95)	61 (84)	0.213	32 (89)	24 (86)	0.703
≥ 0.1%	3 (6)	5 (33)		1 (5)	12 (16)		4 (11)	4 (14)	

Italic signifies *P* < 0.05

### Associations between immunophenotypic markers and genetic abnormalities

Patients with *KMT2A* rearrangements showed a statistically significant lower frequency of CD13, CD33, CD22 and CD34 expression, and higher frequency of CD7, NG2, CD15 and CD133 expression. The high frequency of CD15 and CD133 in *KMT2A*-rearranged subgroup was found to be due to the expression in patients with *KMT2A*-*AFF1*^+^, and was not found in other *KMT2A*-rearranged patients. Frequency in the latter was similar to that in non-*KMT2A*-rearranged patients. Also, CD2, CD5 and CD66c expression was not detected in any of the *KMT2A*-rearranged patients, although no statistically significant differences were found (see Table 3). In addition, no correlations were found between immunological markers and other genetic abnormalities, including *BCR*-*ABL1*, *ETV6*-*RUNX1* and *IKZF1* deletion (see Additional file 2: Table S8).

**Table 3:** Correlations between *KMT2A* gene rearrangement and immunological markers

Immunological marker	*KMT2A* rearrangement			*KMT2A subgroups*		
	Neg (%)	Pos (%)	*P*	*KMT2A*-*AFF1*^+^ (%)	Other *KMT2A*^+^ (%)	*P*
CD2						
Neg	83 (93)	32 (100)	0.132	16 (100)	16 (100)	–
Pos	6 (7)	0 (0)		0 (0)	0 (0)	
CD5						
Neg	86 (97)	32 (100)	0.293	16 (100)	16 (100)	–
Pos	3 (3)	0 (0)		0 (0)	0 (0)	
CD7						
Neg	86 (97)	25 (78)	*0.001*	12 (75)	13 (81)	0.669
Pos	3 (3)	7 (22)		4 (25)	3 (19)	
CD20						
Neg	78 (94)	26 (94)	0.999	13 (100)	13 (87)	0.484
Pos	5 (6)	2 (6)		0 (0)	2 (13)	
CD22						
Neg	16 (28)	12 (75)	*0.011*	8 (73)	4 (44)	0.362
Pos	41 (72)	8 (25)		3 (27)	5 (56)	
CD56						
Neg	46 (84)	18 (86)	0.999	8 (80)	10 (91)	0.586
Pos	9 (16)	3 (14)		2 (20)	1 (9)	
CD58						
Neg	45 (82)	16 (76)	0.581	8 (80)	8 (73)	0.696
Pos	10 (18)	5 (24)		2 (20)	3 (27)	
CD13						
Neg	65 (73)	31 (97)	*0.004*	15 (94)	16 (100)	0.999
Pos	24 (27)	1 (3)		1 (6)	0 (0)	
CD33						
Neg	34 (38)	26 (81)	*<* *0.001*	15 (94)	11 (69)	0.172
Pos	55 (62)	6 (19)		1 (6)	5 (31)	
CD15						
Neg	50 (91)	10 (48)	*<* *0.001*	1 (10)	9 (82)	*0.002*
Pos	5 (9)	11 (52)		9 (90)	2 (18)	
CD66c						
Neg	50 (91)	21 (100)	0.153	10 (100)	11 (100)	–
Pos	5 (9)	0 (0)		0 (0)	0 (0)	
CD34						
Neg	9 (19)	17 (53)	*<* *0.001*	6 (38)	11 (69)	0.077
Pos	80 (90)	15 (47)		10 (62)	5 (31)	
CD133						
Neg	39 (71)	9 (43)	*0.023*	1 (10)	8 (73)	*0.008*
Pos	16 (29)	12 (57)		9 (90)	3 (27)	
NG2						
Neg	43 (78)	3 (14)	*<* *0.001*	0 (0)	3 (73)	0.214
Pos	12 (22)	18 (86)		10 (100)	8 (27)	

Italic signifies *P* < 0.05

### Outcomes and prognostic factors analysis

**Fig. 1:**
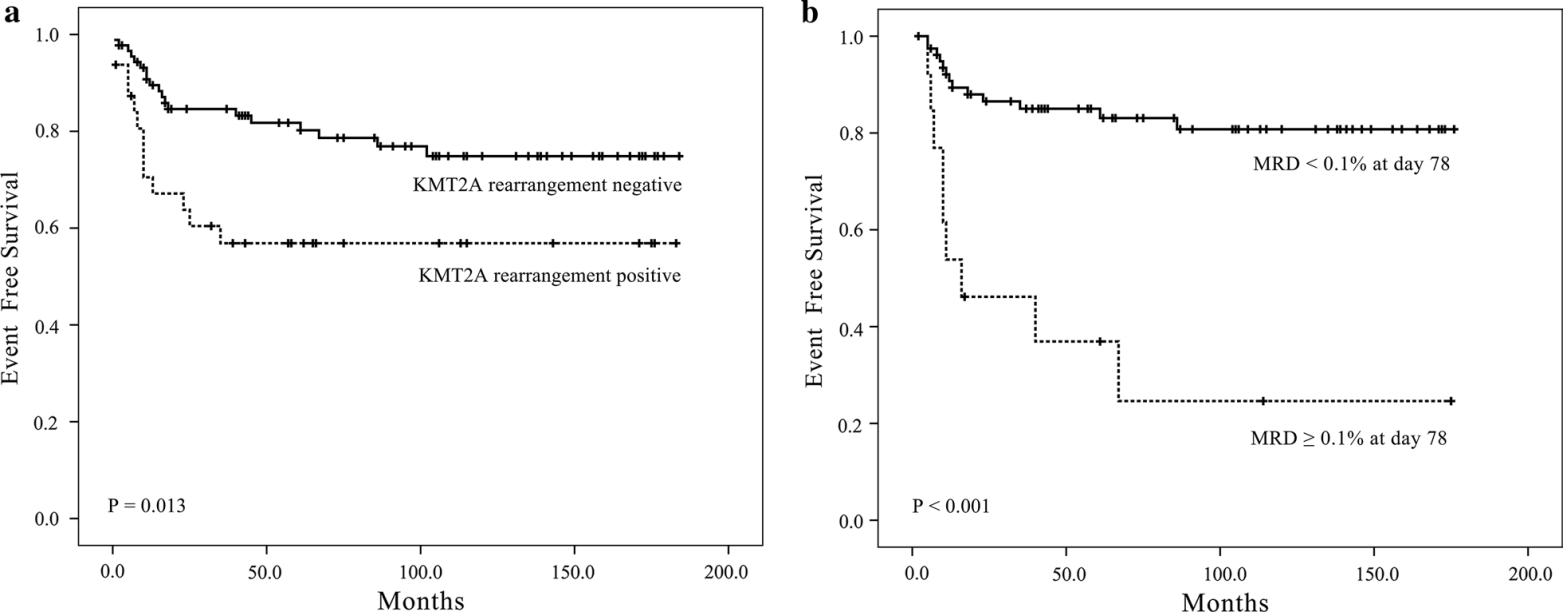
Event-free survival (EFS) for pediatric pro-B ALL according to *KMT2A* rearrangements and minimal residual disease (MRD). **a** EFS stratified by *KMT2A* rearrangements. **b** EFS stratified by MRD at day 78

Follow-up of these 121 pediatric pro-B-ALL patients ranged from 0.5 to 184.0 months (median, 66.0 months), with a 5-year EFS of 75.1 ± 4.1%. During follow-up, one patient had induction failure, twenty-three patients experienced bone marrow relapse, four patients experienced isolated extramedullary relapse, three patients died of severe infections during induction, one patient died of severe toxicity from high-dose methotrexate (HD-MTX) and 89 patients were in first remission. Because the CCLG-2008 protocol introduced MRD evaluation to modify treatment intensity, comparison of outcomes revealed no significant differences between protocols: 5-year EFS was 73.9 ± 6.5% for BCH-2003 and 76.1 ± 5.3% for CCLG-2008, *P *= 0.975. However, the EFS rates varied significantly between clinical-biological subgroups. Favorable outcomes were found in patients older than 1 year, those with absence of CD20 expression and with *KMT2A* rearrangements, and with an MRD lower than 1% at day 33 and 0.1% at day 78, as shown in Table 4. However, other clinical and biological factors were not associated with patients’ outcomes (see Additional file 2: Table S8). Cox proportional hazards regression analysis identified MRD ≥ 0.1% at day 78 and presence of *KMT2A* gene rearrangements as independent adverse prognostic factors (OR 9.424 [95% CI 3.210, 27.662; *P *< 0.001); and (OR 4.142 CI 1.535, 11.715; *P *= 0.005); respectively (see Table 4 and Fig. 1). In the analysis of the outcomes of patients without any fusion, MRD at day 33 and day 78 were also found to be associated with patients’ outcomes, but only MRD at day 78 was an independent prognostic factor; the other biological characteristics were not associated with outcomes. (see Additional file 2: Tables S9, S10 and Additional file 6: Figure S5).

**Table 4:** Prognostic indicators of pro-B-ALL

Clinico-biological features	Event-free survival		Multivariate analysis	
	Survival rate (%)	*P*	HR [95% CI]	*P*
Age (years)				
< 1	54.5 ± 15.0	*0.042*	–	0.534
≥ 10	87.0 ± 7.0			
1–10	74.5 ± 5.0			
CD20				
Positive	42.9 ± 18.7	*0.034*	–	0.087
Negative	78.9 ± 4.2			
*KMT2A*-*rearranged*				
Positive	56.9 ± 9.1	*0.013*	4.142 [1.535, 11.715]	*0.005*
Negative	81.8 ± 4.3		Reference	
MRD at day 33				
≥ 1%	34.9 ± 13.2	*<* *0.001*	–	0.376
0.01–1%	82.8 ± 5.7			
< 0.01%	84.6 ± 7.1			
MRD at day 78				
≥ 0.1%	36.9 ± 13.8	*<* *0.001*	9.424 [3.210, 27.662]	*<* *0.001*
< 0.1%	85.0 ± 4.2		Reference	

Italic signifies *P* < 0.05

## Discussion

In previous studies, Pro-B immunophenotype was regarded as an unfavorable outcome indicator in both pediatric and adult ALL [2, 4], and patients with pro-B-ALL were considered as candidates for allogeneic hematopoietic stem cell transplantation (allo-HSCT) [3]. However, after gaining an impressive understanding of the associations between genetic abnormalities and treatment effects for ALL, pro-B-ALL was shown to be a heterogeneous malignancy with clinical and biological diversity [3, 18]. In the present study, we investigated the genetic and immunological characteristics of pro-B-ALL and their correlation with outcomes in a large cohort of Chinese pediatric pro-B-ALL patients treated using two consecutive protocols. Four common genetic alterations, including *ETV6*-*RUNX1*, *E2A*-*PBX1*, *BCR*-*ABL1* and *KMT2A* rearrangements, were all present in pro-B-ALL. *KMT2A* rearrangement was the most frequent (~ 26%) translocation, and was lower than those reported previously in childhood (~ 50%) and adult (~ 64–75%) pro-B-ALL patients with Caucasian origins [5, 18–20]. However, the proportion (81.82%, 9/11) of *KMT2A*-rearranged infant pro-B-ALL was similar to that in previous findings [21, 22]. These results suggest that the *KMT2A* gene abnormality plays a leading role in the leukemogenesis mechanism of infant leukemia, regardless of racial differences, whereas the gene profile of Chinese pediatric patients with pro-B-ALL differs in part from those in western countries. In line with other reports [2, 5], *KMT2A*-rearranged patients in the present study also presented with adverse clinical features, including high leukocyte counts and younger age at diagnosis.

In the present study, the detailed analysis of immunological markers showed them to be associated with treatment response, *KMT2A* rearrangements and patients’ outcomes. Patients with *KMT2A* rearrangements were disclosed as having typical immunological profiles, with a lower proportion of CD33^+^, CD13^+^, CD22^+^ and CD34^+^ expression and a higher proportion of CD7^+^, CD15^+^, CD133^+^ and NG2^+^ expression, in which the higher rates of CD15 and CD133 expression were mostly due to *KMT2A*-*AFF1*^+^ rather than other *KMT2A* abnormalities. These results are similar to those of previous reports [6–8]. The blasts of *KMT2A*-rearranged leukemia are commonly regarded as being derived from an immature precursor. In their converse correlation with stem cell marker CD34 expression, *KMT2A*-rearranged blasts are shown to be arrested at a very early stage of stem cell differentiation before CD34 expression. A recent study identified the presence of CD34^−^ leukemia-initiating cells (LIC) in primary *KMT2A*-rearranged ALL samples [23], which may partly explain the clinical findings of the present study. Moreover, CD34^+^ patients had more favorable MRD at day 33 in our study and a good prognosis in other studies [24], which also supports this presumption.

In the present study, we also found that an extremely high rate (90%) of *KMT2A*-*AFF1*^+^ patients had CD133 and CD15 expression. CD133 is documented as a more specific marker of hematopoietic stem cells than CD34, and a marker of cancer stem cells (CSC) discovered in many tumors [25, 26]. Furthermore, Mak et al. [27] reported that AFF1 is a promoter of CD133 transcription and CD133 is required for *KMT2A*-*AFF1*^+^ leukemia cell survival. The correlation between CD15 expression and *KMT2A*-*AFF1* has also been reported [6], but the potential mechanisms have still not been described. In addition, CD15^+^ patients in the present study were associated with high MRD at day 33 and day 78, indicating that the mechanism of CD15 expression in the *KMT2A*-*AFF1* subset must be clarified in order to improve treatment for these patients.

NG2 was reported to be expressed in malignant hematopoietic cells and absent in normal hematopoietic cells, thus it could possibly be used as a marker for monitoring MRD and as a potential therapeutic target [28]. A recent study demonstrated that NG2 expression was associated with CNS disease and relapse in *KMT2A*-rearranged infant B-ALL [29]. Also, the combination of anti-NG2-specific monoclonal antibody and chemotherapy improved the prognosis of NSG mice [30]. In the present study, we also found a close association between NG2 expression and *KMT2A* rearrangements, which implies that anti-NG2 antibody may be a potential component in the combination treatment of *KMT2A*-rearranged patients.

In the prognostic analysis of the present study, CD20 expression was found to be associated with unfavorable outcomes in the pro-B-ALL cohort. Although the prognostic impact of CD20 expression on patients’ outcomes remains controversial [31–38], our data suggest that the combination of rituximab with current chemotherapy may be necessary for these patients. In addition, carrying *KMT2A* rearrangement and high MRD at day 78 were shown to be independent prognostic factors of pro-B-ALL in the present study. This emphasizes that genetic detection and MRD monitoring are essential in the prediction of patients’ prognosis. However, we noted that the EFS did not increase, even though we applied the high-risk chemotherapy regimen on *KMT2A*-rearranged patients and enhanced the treatment intensity based on MRD analysis in patients on the CCLG-2008 protocol. Thus, to further improve the treatment outcomes of pro-B-ALL patients, it appears to be necessary to apply new target drugs in combination with current therapy.

## Conclusions

Results of the present study conducted in a large cohort of Chinese pediatric patients treated in a single institution confirm and extend previous reports of the heterogeneity of pro-B-ALL. Moreover, our results demonstrated the importance of genetic investigation and MRD monitoring in prognostic evaluation. However, conventional intensive chemotherapy alone did not improve patients’ long-term outcomes, thus further investigation is necessary to better elucidate the effects of combination therapy with targeted agents or HSCT for *KMT2A*-rearranged in a large cohort of high-risk patients.

## **Supplementary information**

**Additional file 1: Figure S1.** BCH-2003 and CCLG-2008 treatment protocol. (A) BCH-2003 treatment protocol. (B) CCLG-2008 treatment protocol. BCH, Beijing Children’s Hospital; CCLG, Chinese Childhood Leukemia Group; VDLP, vincristine, duanorubicin, l-asparaginase, prednisone; CAM, cyclophosphamide, cytarabine, 6-mercaptopurine; HD-MTX, high-dose methotrexate; VDLD, vincristine, daunorubicin, l-asparaginase, dexzmethasone; VD, vincristine, dexamethasone; IT, intrathecal injection with dexamethasone and methotrexate; VDLA, vincristine, cytarabine, l-asparaginase, dexamethasone; VM26, teniposide; HD-Ara-C, high-dose cytarabine; CA, cyclophosphamide, cytarabine; TIT, intrathecal injection with dexamethasone, methotrexate and cytarabine; I’, Berlin-Frankfürt-Münster (BFM) High Risk block-1’; II’, BFM High Risk block-2’; III’, BFM High Risk block-3’; TP1, minimal residual disease (MRD) time point 1 at the end of induction; TP2, MRD time point 2 before consolidation.

**Additional file 2: Table S1.** Stratification criteria for BCH-2003 and CCLG-2008 treatment protocol. **Table S2.** Immunophenotyping Panel. **Table S3.** Immunophenotypic combinations used in MRD detection in B-ALL. **Table S4.** Incidence of fusion transcript of pro-B ALL. **Table S5.** Comparison of clinical features between *KMT2A* rearranged subgroups. **Table S6.** Expression frequency of immunological markers. **Table S7.** Correlation of immunological markers with clinical features of pro-B ALL. **Table S8.** Correlation of genetic abnormalities with immunological markers. **Table S9.** Associations between patient outcomes and clinical-biological characteristics. **Table S10.** Prognostic indicators of pro-B ALL without any fusion.

**Additional file 3: Figure S2.** Immunophenotyping of a patient with pro-B ALL. Cells in the R2 region express: CD33, CD34, HLA-DR, CD19 and cyCD79a. Under the EGIL criteria, the immunophenotype is pro-B ALL with myeloid marker (CD33).

**Additional file 4: Figure S3.** Representative FISH analysis of KMT2A rearrangement in a patient with pro-B ALL. *KMT2A* gene rearrangement is positive using a KMT2A break-apart probe.

**Additional file 5: Figure S4.** Screening of immunophenotypic markers of minimal residual disease (MRD) monitoring of a patient with pro-B ALL. CyTdT, CD38, CD45, CD15, CD58, CD56, CD133 and NG2 were positive on the leukemic cells.

**Additional file 6: Figure S5.** Event-free survival (EFS) for pediatric pro-B ALL without any fusion according to minimal residual disease (MRD). (A) EFS stratified by MRD at day 33. (B) EFS stratified by MRD at day 78.

## Data Availability

The datasets used and/or analyzed during this study are available from the corresponding authors on reasonable request.

## Abbreviations

Pro-B-ALL: B cell precursor acute lymphoblastic leukemia
CNS: central nervous system
MRD: minimal residual disease
EFS: event free survival
BCH-2003: Beijing Children’s Hospital-2003
CCLG-2008: Chinese Childhood Leukemia Group-2008
SR: standard risk
IR: intermediate risk
HR: high risk
EGIL: European Group for the Immunological Characterization of Acute Leukemia
FISH: fluorescence in situ hybridization
RT-PCR: reverse transcription polymerase chain reaction
HD-MTX: high dose methotrexate
CI: confidence interval
allo-HSCT: allogeneic hematopoietic stem cell transplantation
LIC: leukemia initiating cell
CSC: cancer stem cell
