# Supplementary figures and images for: Clinical-biological characteristics and treatment outcomes of pediatric pro-B ALL patients enrolled in BCH-2003 and CCLG-2008 protocol: a study of 121 Chinese children
Source: Cancer Cell Int. 2019 Nov 14;19:293. doi: 10.1186/s12935-019-1013-9 (PMC6857296; doi:10.1186/s12935-019-1013-9)

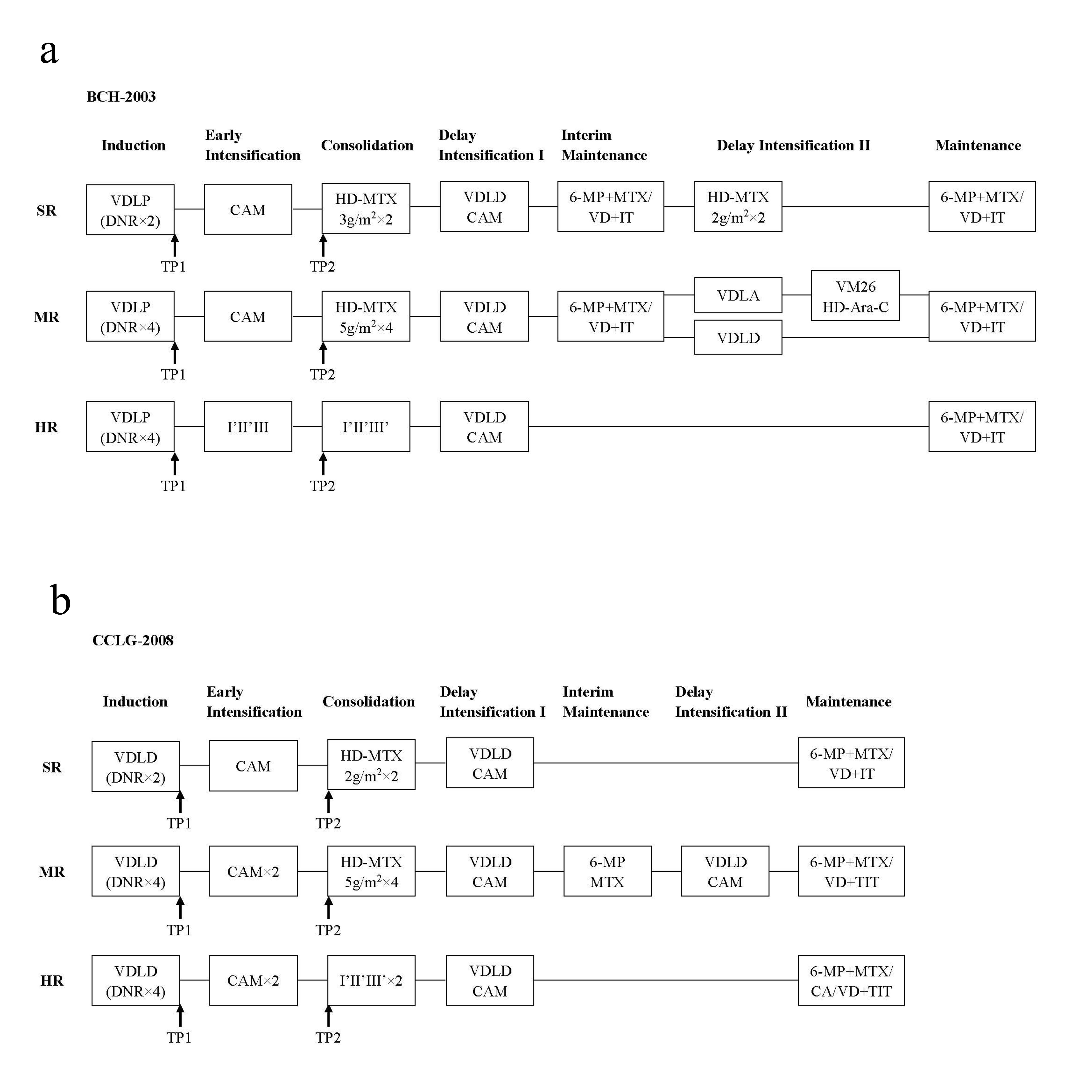

Supplement: Supplementary file 1 — Additional file 1: Figure S1. BCH-2003 and CCLG-2008 treatment protocol. (A) BCH-2003 treatment protocol. (B) CCLG-2008 treatment protocol. BCH, Beijing Children’s Hospital; CCLG, Chinese Childhood Leukemia Group; VDLP, vincristine, duanorubicin, l-asparaginase, prednisone; CAM, cyclophosphamide, cytarabine, 6-mercaptopurine; HD-MTX, high-dose methotrexate; VDLD, vincristine, daunorubicin, l-asparaginase, dexzmethasone; VD, vincristine, dexamethasone; IT, intrathecal injection with dexamethasone and methotrexate; VDLA, vincristine, cytarabine, l-asparaginase, dexamethasone; VM26, teniposide; HD-Ara-C, high-dose cytarabine; CA, cyclophosphamide, cytarabine; TIT, intrathecal injection with dexamethasone, methotrexate and cytarabine; I’, Berlin-Frankfürt-Münster (BFM) High Risk block-1’; II’, BFM High Risk block-2’; III’, BFM High Risk block-3’; TP1, minimal residual disease (MRD) time point 1 at the end of induction; TP2, MRD time point 2 before consolidation. [file 12935_2019_1013_MOESM1_ESM.jpg]

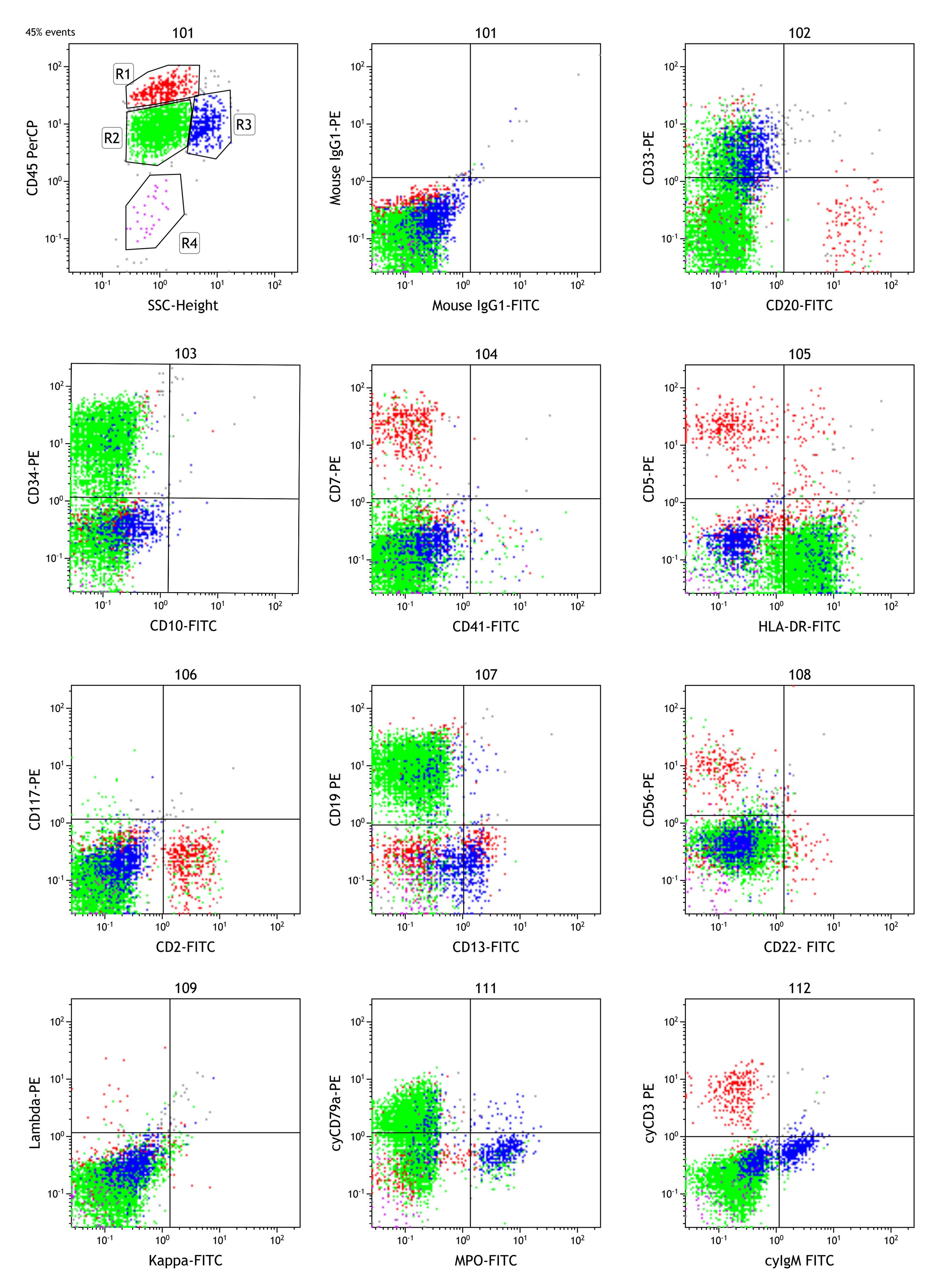

Supplement: Supplementary file 3 — Additional file 3: Figure S2. Immunophenotyping of a patient with pro-B ALL. Cells in the R2 region express: CD33, CD34, HLA-DR, CD19 and cyCD79a. Under the EGIL criteria, the immunophenotype is pro-B ALL with myeloid marker (CD33). [file 12935_2019_1013_MOESM3_ESM.jpg]

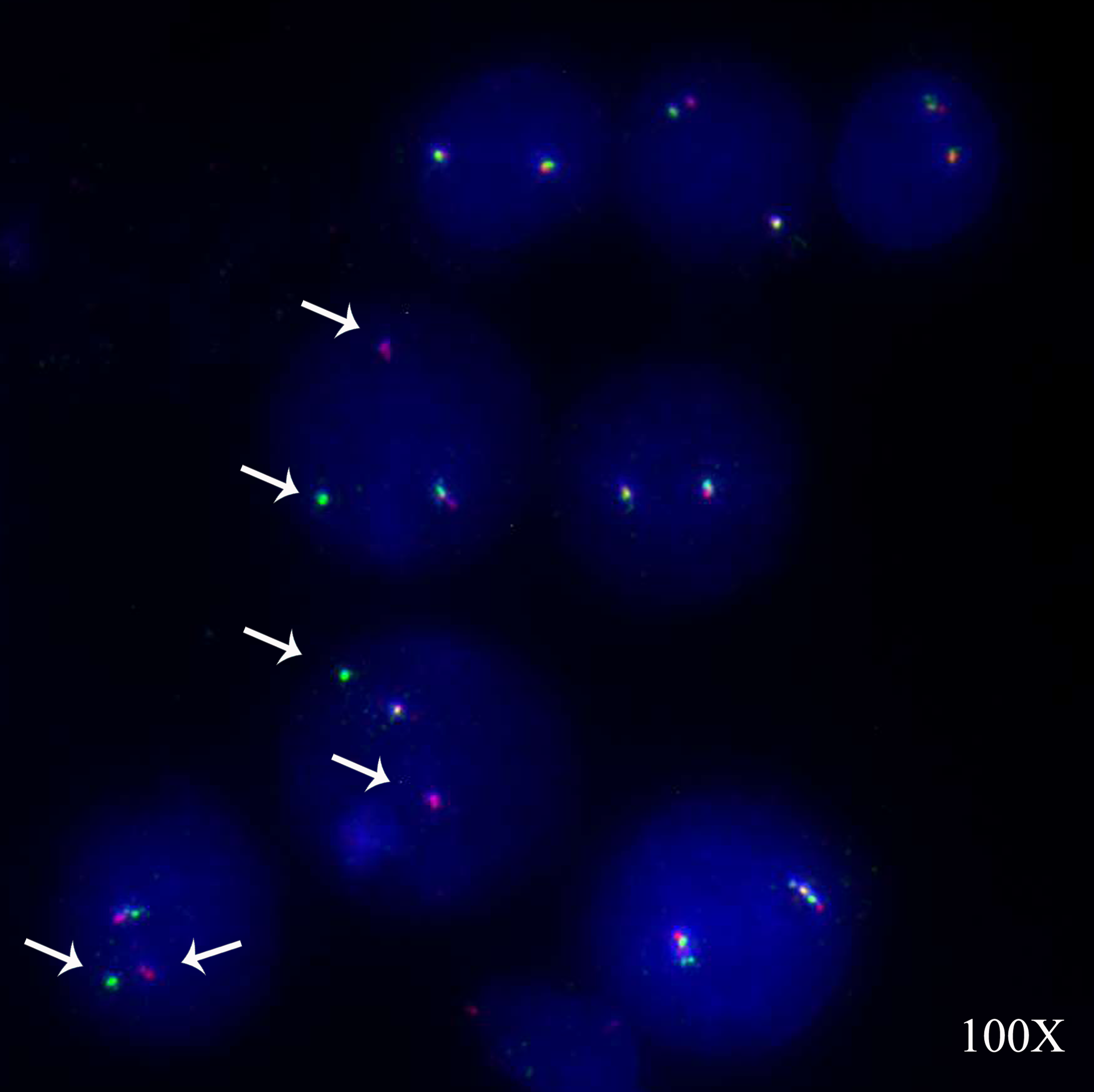

Supplement: Supplementary file 4 — Additional file 4: Figure S3. Representative FISH analysis of KMT2A rearrangement in a patient with pro-B ALL. KMT2A gene rearrangement is positive using a KMT2A break-apart probe. [file 12935_2019_1013_MOESM4_ESM.tif]

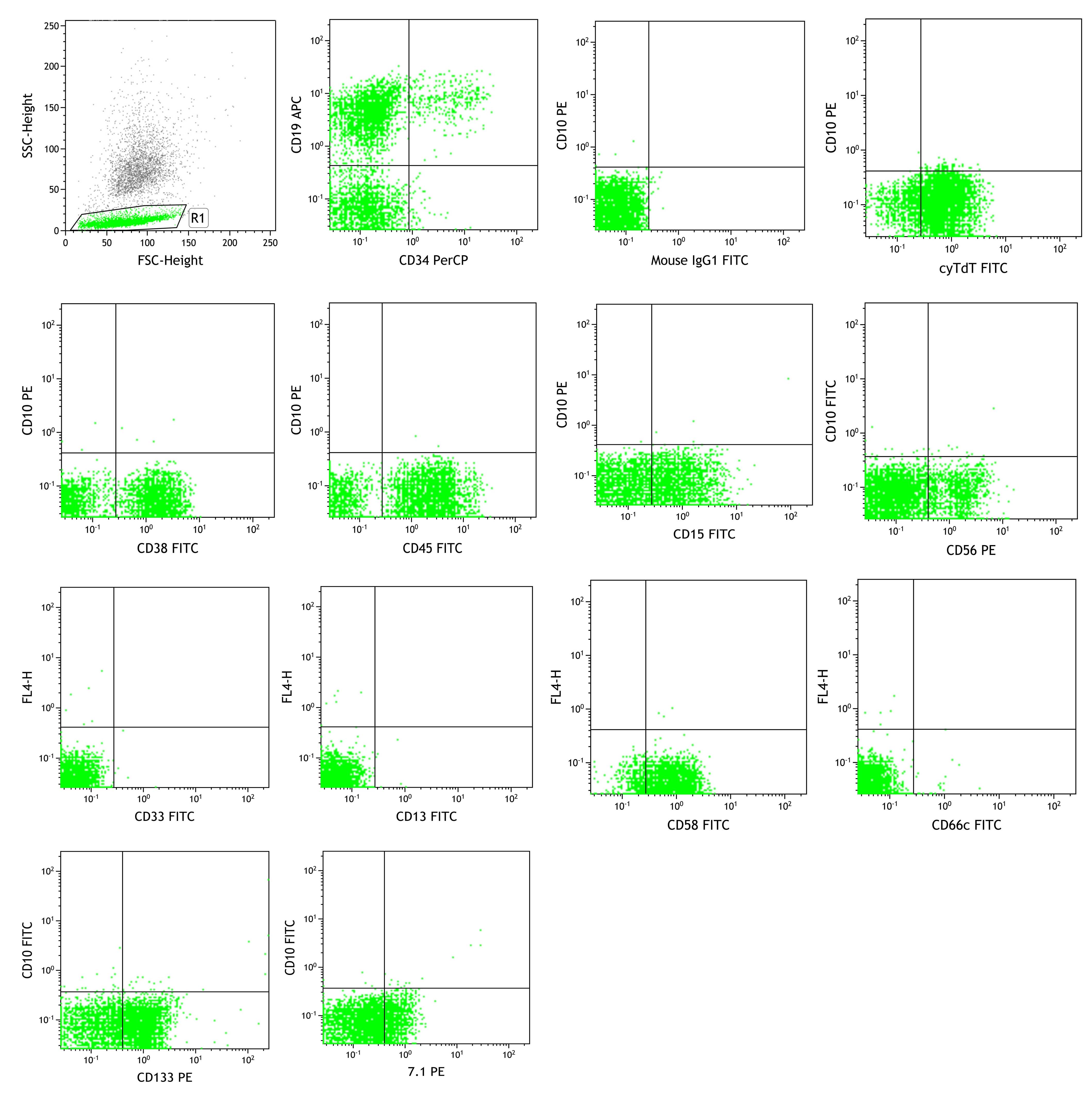

Supplement: Supplementary file 5 — Additional file 5: Figure S4. Screening of immunophenotypic markers of minimal residual disease (MRD) monitoring of a patient with pro-B ALL. CyTdT, CD38, CD45, CD15, CD58, CD56, CD133 and NG2 were positive on the leukemic cells. [file 12935_2019_1013_MOESM5_ESM.jpg]

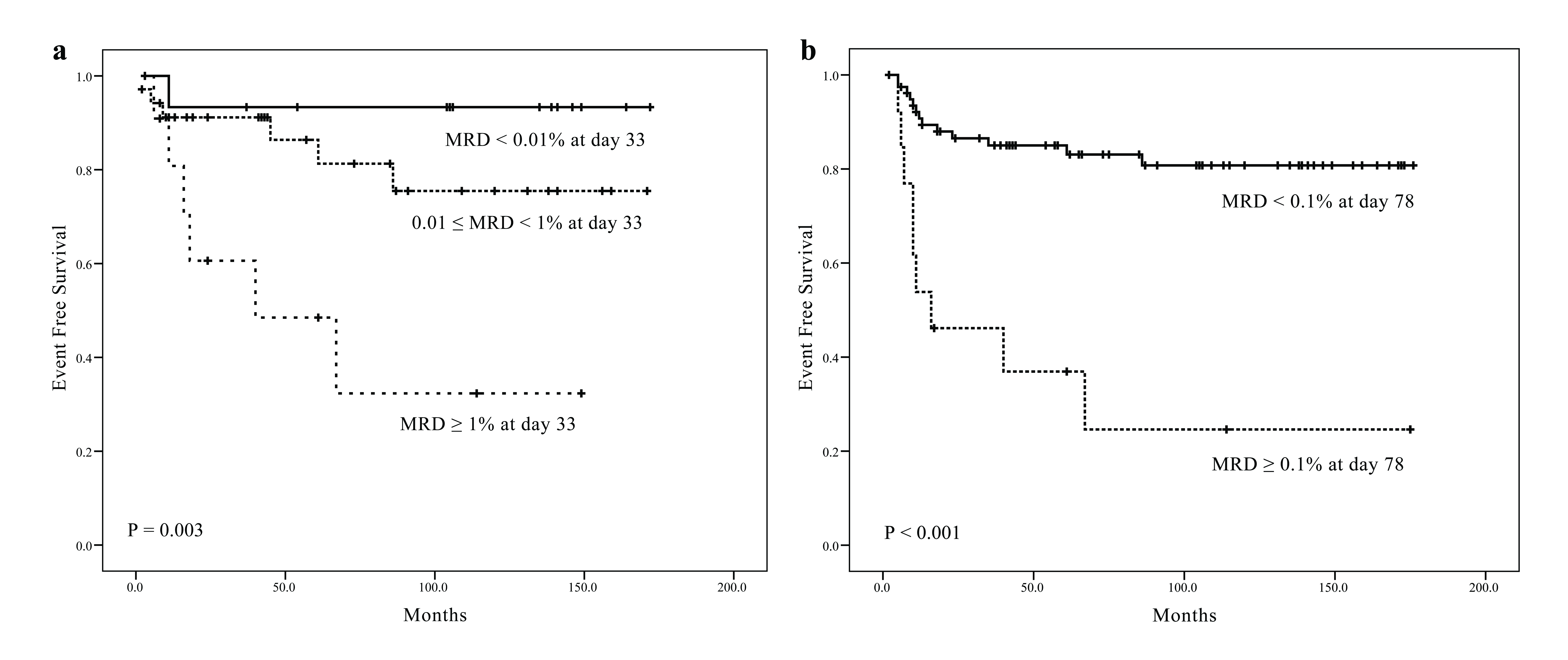

Supplement: Supplementary file 6 — Additional file 6: Figure S5. Event-free survival (EFS) for pediatric pro-B ALL without any fusion according to minimal residual disease (MRD). (A) EFS stratified by MRD at day 33. (B) EFS stratified by MRD at day 78. [file 12935_2019_1013_MOESM6_ESM.tif]
