# Supplementary material for: Clinical-biological characteristics and treatment outcomes of pediatric pro-B ALL patients enrolled in BCH-2003 and CCLG-2008 protocol: a study of 121 Chinese children
Source: Cancer Cell Int. 2019 Nov 14;19:293. doi: 10.1186/s12935-019-1013-9 (PMC6857296; doi:10.1186/s12935-019-1013-9)
Supplement: Supplementary file 2 — Additional file 2: Table S1. Stratification criteria for BCH-2003 and CCLG-2008 treatment protocol. Table S2. Immunophenotyping Panel. Table S3. Immunophenotypic combinations used in MRD detection in B-ALL. Table S4. Incidence of fusion transcript of pro-B ALL. Table S5. Comparison of clinical features between KMT2A rearranged subgroups. Table S6. Expression frequency of immunological markers. Table S7. Correlation of immunological markers with clinical features of pro-B ALL. Table S8. Correlation of genetic abnormalities with immunological markers. Table S9. Associations between patient outcomes and clinical-biological characteristics. Table S10. Prognostic indicators of pro-B ALL without any fusion. [file 12935_2019_1013_MOESM2_ESM.docx]

Table S1. Stratification criteria for BCH-2003 and CCLG-2008 treatment protocol

| Risk group | BCH-2003 | CCLG-2008 |
| --- | --- | --- |
| SR | 1≤age<6  WBC<20×10^9^/L  GPR  No T-cell and not mature B-cell  No t(9;22) or *MLL* rearrangements  BM morphology was M1 at day 33 | 1≤age<10  WBC<50×10^9^/L  GPR  No T-cell and not mature B-cell  No t(9;22), t(1;19) or *MLL* rearrangements  BM morphology was M1/M2 at day15 and M1 at day 33  No CNSL  MRD<10^-4^ at day 33 |
| IR | GPR  No t(9;22)  BM morphology was M1 at day 33  Any one of:  Age ≥6 or <1  WBC ≥20×10^9^/L  T-cell  *MLL* rearrangements | GPR  No t(9;22) or MLL rearrangements  BM morphology at day15 was M1/M2 with IR protocol or M3 with SR protocol  MRD<10^-2^ at day 33 and <10^-3^ at day 78  Any one of:  Age <1  Age ≥10  WBC ≥50×10^9^/L  T-cell  CNSL with no other high-risk factor |
| HR | Any one of:  PPR  t(9;22)  BM morphology was M2/M3 at day 33 | Any one of:  PPR  t(9;22) or MLL rearrangements  BM morphology was M2/M3 at day 33  MRD ≥10^-2^ at day 33  MRD ≥10^-3^ at day 78 |

SR: standard risk; IR: intermediate risk; HR: high risk; WBC: white blood count; GPR: good prednisone response; PPR: poor prednisone response; BM: bone marrow; CNSL: central nervous system leukemia; MRD: minimal residual disease; M1: <5% blasts; M2: 5%≥ blasts <25%; M3: ≥25% blasts.

Table S2 Immunophenotyping Panel

| *Cytomembrane markers panel* | *Cytoplasma markers panel* |
| --- | --- |
| SSC-Height CD45-PerCP | Mouse IgG1-FITC Mouse IgG1-PE |
| Mouse IgG1-FITC Mouse IgG1-PE | MPO-FITC cyCD79a-PE |
| CD20-FITC CD33-PE | cyIgM-FITC cyCD3-PE |
| CD10-FITC CD34-PE |  |
| CD7-PE CD41-FITC |  |
| HLA-DR-FITC CD5-PE |  |
| CD2-FITC CD117-PE |  |
| CD13-FITC CD19-PE |  |
| Ig Kappa-FITC Ig Lambda-PE |  |
| CD22-FITC CD56-PE |  |

Table S3 Immunophenotypic combinations used in MRD detection in B-ALL

| Phenotype combination | Frequency (%) |
| --- | --- |
| CD19/CD34/CD10/TdT | 43.2 |
| CD19/CD34/CD10/CD38 | 67.9 |
| CD19/CD34/CD10/CD45 | 53.1 |
| CD19/CD34/CD10/CD13 | 4.5 |
| CD19/CD34/CD10/CD15 | 3.8 |
| CD19/CD34/CD10/CD33 | 4.5 |
| CD19/CD34/CD10/CD58 | 34.0 |
| CD19/CD34/CD10/CD66C | 30.3 |
| CD19/CD34/CD10/CD56 | 1.6 |
| CD19/CD34/CD10/CD133 | 2.1 |
| CD19/CD34/CD10/NG2 | 2.7 |

Table S4. Incidence of fusion transcript of pro-B ALL

| Fusion transcript | No. of Patients | Incidence (%) |
| --- | --- | --- |
| *ETV6-RUNX1* | 3 | 2.48 |
| *E2A-PBX1* | 1 | 0.82 |
| *BCR-ABL1* | 4 | 3.31 |
| *KMT2A* rearrangements | 32 | 26.45 |
| *KMT2A-AFF1* | 16 | 13.22 |
| *KMT2A-MLLT3* | 6 | 4.96 |
| *KMT2A –MLLT10* | 3 | 2.48 |
| *KMT2A –MLLT1* | 3 | 2.48 |
| *KMT2A –EPS15* | 1 | 0.82 |
| *KMT2A -SEPT6* | 1 | 0.82 |
| *KMT2A - KMT2A* | 1 | 0.82 |
| *KMT2A ^+*^* | 1 | 0.82 |
| Total | 40 | 33.06 |

* This patient was found to have *KMT2A* gene disruption by FISH. However, no partner gene was determined by PCR method.

Table S5. Comparison of clinical features between *KMT2A* rearranged subgroups

| Clinical features | *KMT2A-AFF1*^+^ | Other *KMT2A*^+^ | *P* value |
| --- | --- | --- | --- |
| Gender | | | |
| Male | 7 | 11 | 0.285 |
| Female | 9 | 5 |  |
| Age (years) | | | |
| <1 | 3 | 6 | 0.556 |
| 1~10 | 11 | 9 |  |
| ≥10 | 2 | 1 |  |
| WBC | | | |
| <50×10^9^/L | 6 | 10 | 0.289 |
| ≥50×10^9^/L | 10 | 6 |  |
| Prednisone response | | | |
| Good | 15 | 14 | 1.000 |
| Poor | 1 | 2 |  |
| MRD at day 33 | | | |
| <0.01% | 5 | 5 | 1.000 |
| 0.01%~1% | 7 | 7 |  |
| ≥1% | 2 | 1 |  |
| MRD at day 78 | | | |
| <0.1% | 11 | 11 | 0.342 |
| ≥0.1% | 4 | 1 |  |

Table S6 Expression frequency of immunological markers

| Immunological Marker | No. of positive patients | Total No. of patients detected | Frequency (%) |
| --- | --- | --- | --- |
| CD2 | 6 | 121 | 4.96 |
| CD5 | 3 | 121 | 2.48 |
| CD7 | 10 | 121 | 8.26 |
| CD13 | 25 | 121 | 20.66 |
| CD15 | 16 | 76 | 21.05 |
| CD20 | 7 | 111 | 6.31 |
| CD22 | 49 | 77 | 63.64 |
| CD33 | 61 | 121 | 50.41 |
| CD34 | 95 | 121 | 78.51 |
| CD56 | 12 | 76 | 15.79 |
| CD58 | 15 | 76 | 12.40 |
| CD66c | 5 | 76 | 6.58 |
| CD117 | 3 | 121 | 2.48 |
| CD133 | 28 | 76 | 36.84 |
| NG2 | 30 | 76 | 39.47 |
| cyTdT | 64 | 76 | 84.21 |

Table S7 Correlation of immunological markers with clinical features of pro-B ALL

| Clinical features | CD2 | | | | CD7 | | | | CD20 | | | | CD22 | | | CD56 | | | CD58 | | | CD13 | | | CD33 | | | CD66c | | | CD133 | | |
| --- | --- | --- | --- | --- | --- | --- | --- | --- | --- | --- | --- | --- | --- | --- | --- | --- | --- | --- | --- | --- | --- | --- | --- | --- | --- | --- | --- | --- | --- | --- | --- | --- | --- |
|  | Neg | | Pos | P | Neg | | Pos | P | Neg | | Pos | P | Neg | Pos | P | Neg | Pos | P | Neg | Pos | P | Neg | Pos | P | Neg | Pos | P | Neg | Pos | P | Neg | Pos | P |
| Gender | |  | | | |  | | | |  | | | | | | | | | | | | | | | | | | | | | | | |
| Male | 66 | | 3 | 0.721 | 64 | | 5 | 0.639 | 57 | | 4 | 0.999 | 19 | 26 | 0.237 | 40 | 4 | 0.060 | 35 | 9 | 0.854 | 53 | 16 | 0.429 | 34 | 35 | 0.937 | 36 | 2 | 0.358 | 29 | 15 | 0.560 |
| Female | 49 | | 3 |  | 47 | | 5 |  | 47 | | 3 |  | 9 | 23 |  | 24 | 8 |  | 26 | 6 |  | 43 | 9 |  | 26 | 26 |  | 23 | 3 |  | 19 | 13 |  |
| Age (year) | |  | | | |  | | | |  | | | | | | | | | | | | | | | | | | | | | | | |
| <1 | 11 | | 0 | 0.527 | 81 | | 6 | 0.052 | 10 | | 1 | 0.533 | 0 | 2 | 0.809 | 7 | 3 | 0.393 | 9 | 1 | 0.444 | 11 | 0 | 0.187 | 9 | 2 | 0.080 | 9 | 0 | 0.455 | 6 | 4 | 0.881 |
| 1~10 | 83 | | 4 |  | 22 | | 1 |  | 74 | | 6 |  | 20 | 34 |  | 48 | 8 |  | 43 | 13 |  | 68 | 19 |  | 40 | 47 |  | 40 | 5 |  | 35 | 21 |  |
| ≥10 | 21 | | 2 |  | 8 | | 3 |  | 20 | | 0 |  | 8 | 13 |  | 9 | 1 |  | 9 | 1 |  | 17 | 6 |  | 11 | 12 |  | 10 | 0 |  | 7 | 3 |  |
| WBC | |  | | | |  | | | |  | | | | | | | | | | | | | | | | | | | | | | | |
| <50×10^9^/L | 78 | | 6 | 0.095 | 76 | | 8 | 0.448 | 72 | | 5 | 0.999 | 16 | 36 | 0.206 | 45 | 9 | 0.999 | 44 | 10 | 0.676 | 63 | 21 | 0.076 | 39 | 45 | 0.295 | 41 | 3 | 0.660 | 36 | 18 | 0.320 |
| ≥50×10^9^/L | 37 | | 0 |  | 35 | | 2 |  | 32 | | 2 |  | 12 | 13 |  | 19 | 3 |  | 17 | 5 |  | 33 | 4 |  | 21 | 16 |  | 18 | 2 |  | 12 | 10 |  |
| Prednisone response | | | | | |  | | | |  | | | | | | | | | | | | | | | | | | | | | | | |
| Good | 107 | | 5 | 0.377 | 102 | | 10 | 0.349 | 97 | | 6 | 0.417 | 25 | 45 | 0.700 | 58 | 12 | 0.581 | 58 | 12 | 0.052 | 88 | 24 | 0.462 | 56 | 56 | 0.748 | 54 | 5 | 0.498 | 43 | 27 | 0.286 |
| Poor | 8 | | 1 |  | 9 | | 0 |  | 7 | | 1 |  | 3 | 4 |  | 6 | 0 |  | 3 | 3 |  | 8 | 1 |  | 4 | 5 |  | 5 | 0 |  | 5 | 1 |  |
| MRD at day 33 | |  | | | |  | | | |  | | | | | | | | | | | | | | | | | | | | | | | |
| <0.01% | 27 | | 0 | 0.118 | 24 | | 3 | 0.665 | 22 | | 3 | 0.372 | 8 | 12 | 0.999 | 11 | 1 | 0.350 | 12 | 0 | 0.088 | 22 | 5 | 0.092 | 17 | 10 | 0.254 | 7 | 0 | 0.703 | 8 | 4 | 0.898 |
| 0.01%~1% | 48 | | 5 |  | 50 | | 3 |  | 48 | | 2 |  | 9 | 15 |  | 35 | 10 |  | 32 | 13 |  | 46 | 7 |  | 23 | 30 |  | 39 | 4 |  | 27 | 18 |  |
| ≥1% | 16 | | 0 |  | 15 | | 1 |  | 10 | | 1 |  | 4 | 8 |  | 11 | 1 |  | 10 | 2 |  | 10 | 6 |  | 8 | 8 |  | 11 | 1 |  | 7 | 5 |  |
| MRD at day 78 | |  | | | |  | | | |  | | | | | | | | | | | | | | | | | | | | | | | |
| <0.1% | 74 | | 5 | 0.351 | 72 | | 7 | 0.264 | 68 | | 5 | 0.514 | 16 | 29 | 0.999 | 47 | 9 | 0.999 | 43 | 13 | 0.911 | 64 | 15 | 0.731 | 42 | 37 | 0.639 | 45 | 5 | 0.349 | 34 | 22 | 0.564 |
| ≥0.1% | 13 | | 0 |  | 13 | | 0 |  | 8 | | 1 |  | 4 | 6 |  | 7 | 1 |  | 6 | 2 |  | 10 | 3 |  | 6 | 7 |  | 8 | 0 |  | 4 | 4 |  |

Table S8 Correlation of genetic abnormalities with immunological markers

| Immunological Marker | *BCR-ABL1* | | | |  | *ETV6-RUNX1* | | | | |  | *IKZF1* deletion | | | |
| --- | --- | --- | --- | --- | --- | --- | --- | --- | --- | --- | --- | --- | --- | --- | --- |
|  | Neg | | Pos | P |  | Neg | | Pos | P |  | | | Neg | Pos | P |
| CD2 | |  | | | | |  | | | | | | | | |
| Neg | 111 | | 4 | 0.642 |  | 112 | | 3 | 0.689 |  | | | 67 | 13 | 0.085 |
| Pos | 6 | | 0 |  |  | 6 | | 0 |  |  |  |  | 2 | 2 |  |
| CD5 | |  | | | | |  | | | | | | | | |
| Neg | 114 | | 4 | 0.746 |  | 115 | | 3 | 0.780 |  | | | 67 | 15 | 0.505 |
| Pos | 3 | | 0 |  |  | 3 | | 0 |  |  |  |  | 2 | 0 |  |
| CD7 | |  | | | | |  | | | | | | | | |
| Neg | 107 | | 4 | 0.542 |  | 108 | | 3 | 0.599 |  | | | 64 | 14 | 0.937 |
| Pos | 10 | | 0 |  |  | 10 | | 0 |  |  |  |  | 5 | 1 |  |
| CD20 | |  | | | | |  | | | | | | | | |
| Neg | 102 | | 2 | 0.999 |  | 101 | | 3 | 0.999 |  | | | 64 | 15 | 0.580 |
| Pos | 7 | | 0 |  |  | 7 | | 0 |  |  |  |  | 5 | 0 |  |
| CD22 | |  | | | | |  | | | | | | | | |
| Neg | 27 | | 1 | 0.999 |  | 28 | | 1 | 0.377 |  | | | 14 | 5 | 0.246 |
| Pos | 47 | | 2 |  |  | 48 | | 0 |  |  |  |  | 24 | 3 |  |
| CD56 | |  | | | | |  | | | | | | | | |
| Neg | 61 | | 3 | 0.505 |  | 63 | | 1 | 0.293 |  | | | 40 | 11 | 0.671 |
| Pos | 11 | | 1 |  |  | 11 | | 1 |  |  |  |  | 8 | 1 |  |
| CD58 | |  | | | | |  | | | | | | | | |
| Neg | 57 | | 4 | 0.308 |  | 60 | | 1 | 0.276 |  | | | 38 | 10 | 0.747 |
| Pos | 15 | | 0 |  |  | 14 | | 1 |  |  |  |  | 10 | 2 |  |
| CD13 | |  | | | | |  | | | | | | | | |
| Neg | 93 | | 3 | 0.827 |  | 94 | | 2 | 0.583 |  | | | 58 | 14 | 0.352 |
| Pos | 24 | | 1 |  |  | 24 | | 1 |  |  | | | 11 | 1 |  |
| CD33 | |  | | | | |  | | | | | | | | |
| Neg | 57 | | 3 | 0.301 |  | 59 | | 1 | 0.589 |  | | | 35 | 7 | 0.776 |
| Pos | 60 | | 1 |  |  | 59 | | 2 |  |  |  |  | 34 | 8 |  |
| CD15 | |  | | | | |  | | | | | | | | |
| Neg | 56 | | 4 | 0.289 |  | 58 | | 2 | 0.459 |  | | | 38 | 11 | 0.317 |
| Pos | 16 | | 0 |  |  | 16 | | 0 |  |  |  |  | 10 | 1 |  |
| CD66c | |  | | | | |  | | | | | | | | |
| Neg | 68 | | 3 | 0.127 |  | 69 | | 2 | 0.704 |  | | | 46 | 10 | 0.121 |
| Pos | 4 | | 1 |  |  | 5 | | 0 |  |  |  |  | 2 | 2 |  |
| CD34 | |  | | | | |  | | | | | | | | |
| Neg | 26 | | 0 | 0.287 |  | 24 | | 2 | 0.054 |  | | | 17 | 9 | 0.971 |
| Pos | 91 | | 4 |  |  | 94 | | 1 |  |  |  |  | 52 | 28 |  |
| CD133 | |  | | | | |  | | | | | | | | |
| Neg | 44 | | 4 | 0.117 |  | 46 | | 2 | 0.274 |  | | | 26 | 10 | 0.065 |
| Pos | 28 | | 0 |  |  | 28 | | 0 |  |  |  |  | 22 | 2 |  |
| NG2 | |  | | | | |  | | | | | | | | |
| Neg | 42 | | 4 | 0.097 |  | 44 | | 2 | 0.247 |  | | | 25 | 10 | 0.058 |
| Pos | 30 | | 0 |  |  | 30 | | 0 |  |  |  |  | 23 | 2 |  |

Table S9. Associations between patient outcomes and clinical-biological characteristics

| Variables | All patients | |  | Patients without any fusion | |
| --- | --- | --- | --- | --- | --- |
|  | EFS (%) | *P* |  | EFS | *P* |
| Gender | | | | | |
| Male | 73.3±5.6 | 0.559 |  | 81.4±6.0 | 0.840 |
| Female | 77.5±6.0 |  |  | 80.8±7.1 |  |
| Age (year) | | | | | |
| <1 | 54.5±15.0 | **0.042** |  | 50.0±35.4 | 0.213 |
| 1~10 | 74.5±5.0 |  |  | 80.7±5.3 |  |
| ≥10 | 87.0±7.0 |  |  | 87.5±8.3 |  |
| WBC | | | | | |
| <50×10^9^/L | 78.1±4.7 | 0.237 |  | 78.3±5.6 | 0.354 |
| ≥50×10^9^/L | 68.0±8.0 |  |  | 89.4±7.1 |  |
| CD2 | | | | | |
| Neg | 74.7±4.2 | 0.660 |  | 81.0±4.8 | 0.828 |
| Pos | 83.3±15.2 |  |  | 83.3±15.2 |  |
| CD5 | | | | | |
| Neg | 74.4±4.2 | 0.291 |  | 80.4±4.7 | 0.326 |
| Pos | 100 |  |  | 100 |  |
| CD7 | | | | | |
| Neg | 76.5±4.2 | 0.291 |  | 80.4±4.7 | 0.326 |
| Pos | 60.0±15.0 |  |  | 100 |  |
| CD20 | | | | | |
| Neg | 78.9±4.2 | **0.034** |  | 82.9±4.7 | 0.310 |
| Pos | 42.9±18.7 |  |  | 60.0±21.9 |  |
| CD22 | | | | | |
| Neg | 66.9±9.7 | 0.400 |  | 83.9±10.4 | 0.773 |
| Pos | 79.8±6.1 |  |  | 85.1±6.2 |  |
| CD56 | | | | | |
| Neg | 75.0±5.9 | 0.999 |  | 82.4±6.8 | 0.157 |
| Pos | 73.3±13.2 |  |  | 57.1±18.7 |  |
| CD58 | | | | | |
| Neg | 72.1±6.3 | 0.214 |  | 73.1±8.0 | 0.080 |
| Pos | 85.6±9.5 |  |  | 100 |  |
| CD13 | | | | | |
| Neg | 74.0±4.7 | 0.892 |  | 81.2±5.4 | 0.486 |
| Pos | 79.3±8.2 |  |  | 81.1±8.5 |  |
| CD33 | | | | | |
| Neg | 66.5±6.3 | 0.122 |  | 73.2±8.7 | 0.531 |
| Pos | 83.7±5.0 |  |  | 85.3±5.2 |  |
| CD15 | | | | | |
| Neg | 75.7±6.0 | 0.841 |  | 79.0±6.8 | 0.991 |
| Pos | 71.8±12.0 |  |  | 75.0±21.7 |  |
| CD66c | | | | | |
| Neg | 75.2±5.5 | 0.924 |  | 77.3±6.9 | 0.340 |
| Pos | 66.7±27.2 |  |  | 100 |  |
| CD34 | | | | | |
| Neg | 63.9±9.7 | 0.307 |  | 83.3±15.2 | 0.627 |
| Pos | 78.5±4.4 |  |  | 80.9±4.8 |  |
| CD133 | | | | | |
| Neg | 76.5±6.6 | 0.861 |  | 78.1±8.0 | 0.920 |
| Pos | 73.6±8.6 |  |  | 80.8±10.0 |  |
| NG2 | | | | | |
| Neg | 73.5±7.0 | 0.479 |  | 78.1±7.6 | 0.675 |
| Pos | 77.7±8.0 |  |  | 81.8±11.6 |  |
| Prednisone response | | | | | |
| Good | 74.1±4.3 | 0.780 |  | 79.8±4.9 | 0.817 |
| Poor | 88.9±10.5 |  |  | 100 |  |

Table S10 Prognostic indicators of pro-B ALL without any fusion

| Clinico-biological features | Event-free survival | | |  | | Multivariate analysis | | |
| --- | --- | --- | --- | --- | --- | --- | --- | --- |
|  | Survival Rate (%) | *P* |  | | HR[95%CI] | | *P* |  |
| MRD at day 33 |  |  |  | |  | |  |  |
| ≥10^-2^ | 48.5±16.4 | **<0.001** |  | | / | | 0.137 |  |
| 10^-4^~10^-2^ | 86.4±6.6 |  |  | |  |  |  |  |
| <10^-4^ | 93.3±6.4 |  |  | |  |  |  |  |
| MRD at day 78 |  |  |  | |  | |  |  |
| ≥10^-3^ | 46.9±18.7 | **<0.001** |  | | 7.305[2.175, 24.537] | | **0.001** |  |
| <10^-3^ | 91.6±4.0 |  |  | | Reference | |  |  |
